# Toward Cell‐Specific Molecular MRI: Modulating Chemical Exchange Saturation Transfer Contrast With Manganese

**DOI:** 10.1002/anie.1291856

**Published:** 2026-09-09

**Authors:** Eleonora Cavallari, Valeria Bitonto, Morgana Giachello, Angelo Bifone

**Affiliations:** ^1^ Department of Molecular Biotechnology and Health Sciences University of Torino Torino Italy

**Keywords:** cell specificity, CEST, manganese, MEMRI, metabolic imaging

## Abstract

Chemical exchange saturation transfer (CEST) MRI enables sensitive detection of low‐concentration metabolites in vivo, but its biological interpretation is limited by the composite nature of the signal, which reflects contributions from multiple cell types. In the brain, these include intracellular metabolites in neurons and glial cells, as well as semisolid macromolecular pools. Here, we introduce MangaCEST, a strategy that uses manganese (Mn^2^^+^)‐induced intracellular relaxation changes to modulate the contributions of different cell populations to the CEST spectrum. Mn^2^^+^ enters the cytoplasm via calcium‐permeable channels and accumulates in excitable cells in an activity‐dependent manner, shortening water T_1_ and selectively reducing CEST contrast. Comparison of CEST Z‐spectra with or without Mn^2^^+^ enables MangaCEST to isolate Mn‐sensitive signal components and differentially analyze intracellular contributions. To provide a cell‐type‐weighted Mn^2^^+^ effect in a well‐defined biological framework, we focused on the cerebellum, where preferential neuronal accumulation of Mn^2^^+^ is well documented. In the developing chick embryo in vivo, Mn^2^^+^ caused significant T_1_ shortening (∼28%) and frequency‐dependent CEST spectrum changes. Differential spectra showed attenuation at 3–4 ppm, consistent with contributions from neuronal‐associated metabolite pools. These findings suggest that MangaCEST may represent a promising strategy for disentangling cellular contributions to molecular MRI signals in the brain.

## Graphical abstract

Manganese‐modulated chemical exchange saturation transfer (CEST) MRI, Manga CEST, leverages intracellular Mn^2^^+^ uptake to selectively perturb CEST contrast. Comparing Z‐spectra before and after Mn reveals compartment‐specific contributions. In vivo, neuron‐enriched Mn accumulation induces frequency‐dependent attenuation (3–4 ppm), enabling cell‐weighted molecular imaging beyond conventional CEST.

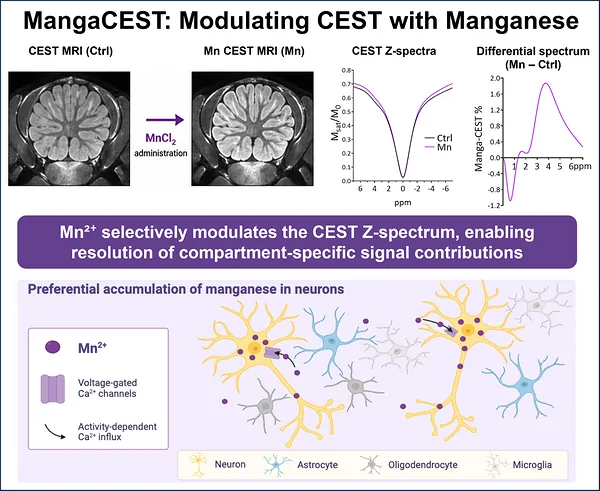

Chemical exchange saturation transfer (CEST) magnetic resonance imaging has emerged as a powerful approach for probing molecular processes in vivo by indirectly detecting low‐concentration metabolites through proton exchange with bulk water [1, 2, 3]. By exploiting exchangeable protons in endogenous compounds such as amides, amines, and hydroxyl groups, CEST enables the mapping of protein content, metabolite distributions, and tissue pH with sensitivity beyond conventional MR spectroscopy [4, 5, 6, 7]. In the brain, CEST has shown considerable promise for the study of metabolism and microenvironmental changes. However, a fundamental limitation remains: CEST contrast reflects a superposition of signals arising from multiple cellular compartments, primarily neurons and glial cells, preventing direct attribution of spectral features to specific cell types.

Here, we introduce MangaCEST, a strategy aimed at selectively modulating the contribution of intracellular compartments to the CEST signal using a paramagnetic perturbation. Our approach leverages the biological properties of manganese ions (Mn^2^^+^), which enter excitable cells through calcium‐permeable channels and exhibit preferential accumulation in neurons and glia [8, 9, 10]. This activity‐dependent intracellular accumulation induces shortening of intracellular water *T*_1_, thereby altering the efficiency of saturation transfer processes associated with the affected compartments.

Rather than directly labeling specific metabolites or cell types, MangaCEST relies on comparing CEST spectra of endogenous proton pools acquired with and without Mn^2^^+^ administration to identify Mn‐sensitive components of the signal. This differential approach provides a means to selectively attenuate the contribution of compartments that preferentially accumulate Mn^2^^+^, that is, cells with higher activity‐dependent calcium influx, thereby enabling a decomposition of the different cellular contributions.

CEST with gadolinium‐based contrast agents (GBCAs) has recently been proposed to probe transmembrane water exchange [11]. In those approaches, gadolinium‐based contrast agents remain confined to the extracellular space and modulate intracellular CEST contrast indirectly via water exchange across the cell membrane, providing sensitivity to membrane permeability and water cycling dynamics [12]. By contrast, MangaCEST operates through a fundamentally different mechanism: direct intracellular accumulation of a paramagnetic agent within excitable cells, and therefore reports on the relative contributions of different cell types, rather than inter‐compartmental exchange. These approaches are thus complementary: GBCA‐CEST interrogates water transport, whereas MangaCEST enables weighing the CEST signal by its cellular constituents.

We demonstrate the feasibility of MangaCEST in vivo using the developing chick embryo in ovo. Systemic Mn^2^^+^ administration has long been exploited in MRI to enhance brain cytoarchitecture [13, 14] and to interrogate neuronal activity via manganese‐enhanced MRI (MEMRI). However, the attainment of biologically relevant intracellular Mn^2^^+^ levels in the brain generally necessitates bypassing the blood–brain barrier (BBB) [15, 16, 17, 18, 19]. At late embryonic stages, the avian brain is already structurally organized, yet the BBB remains immature, allowing systemically administered agents to access the brain parenchyma [11, 20]. This unique feature enables efficient delivery of manganese into the central nervous system without the need for invasive procedures. The model therefore provides a controlled framework to modulate intracellular manganese levels and directly assess their impact on CEST contrast.

Importantly, manganese uptake is not strictly confined to neurons and may depend on multiple physiological factors [21, 22]. To test the proposed approach, we focus on the cerebellum, a brain region in which manganese has been shown to predominantly accumulate in neuronal populations, particularly Purkinje cells, relative to glial compartments [23]. By comparing CEST spectra acquired with and without manganese, we investigate differential spectral features that may be consistent with neuron‐enriched contributions to provide a first proof of concept for the MangaCEST approach.

All MRI experiments were conducted on chick embryos at embryonic day 17 (E17). Two groups were studied (*n* = 5 embryos per group): untreated controls and Mn‐injected embryos receiving 28.5 mg/kg Mn^2^^+^. The choice of E17, in relation to the specific chicken strain and egg size, is further justified in the Supporting Information.

MRI experiments were performed at 7 T using a Bruker Pharmascan system equipped with a 72 mm quadrature birdcage coil. Anatomical localization was achieved using T_2_‐weighted fast spin echo imaging, followed by localized shimming and flip‐angle calibration over the brain. CEST imaging was performed using a RARE sequence with a 3 s continuous saturation pulse (*B*_1_ = 3 µT) and frequency offsets spanning −100 to +100 ppm; a total of 53 offsets were sampled, nonuniformly distributed with higher density in spectral regions of interest (ROI). The water signal was thus measured as a function of the saturation frequency offset relative to the water resonance (set to 0 ppm), yielding the so‐called Z‐spectrum. Exchangeable proton pools selectively reduce the water signal at their characteristic frequency offsets, giving rise to distinct features in the Z‐spectrum. Because these pools are predominantly located at positive frequency offsets relative to water, while the negative offsets are influenced by nuclear Overhauser enhancement (NOE) and magnetization transfer (MT) effects, the Z‐spectrum is generally asymmetric around the water resonance. This asymmetry is commonly quantified using the magnetization transfer ratio asymmetry (MTR_asym), defined as the difference between normalized signals acquired at symmetric positive and negative frequency offsets.

Quantitative *T*_1_ maps were acquired using a saturation recovery RARE sequence with multiple repetition times. Examples of *T*_1_ maps for control and Mn‐injected chicks are provided in the Supplementary Information section. All datasets were coregistered. The region of interest (ROI) employed for MRI image analysis was selected to represent the cerebellum of the chick embryo. As a reference, we also analyzed the effects of manganese in the pallium, a major component of the telencephalon that presents a predominantly nuclear, rather than laminar, cytoarchitecture [24, 25]. The two ROIs are shown in Figure 1.

**FIGURE 1:**
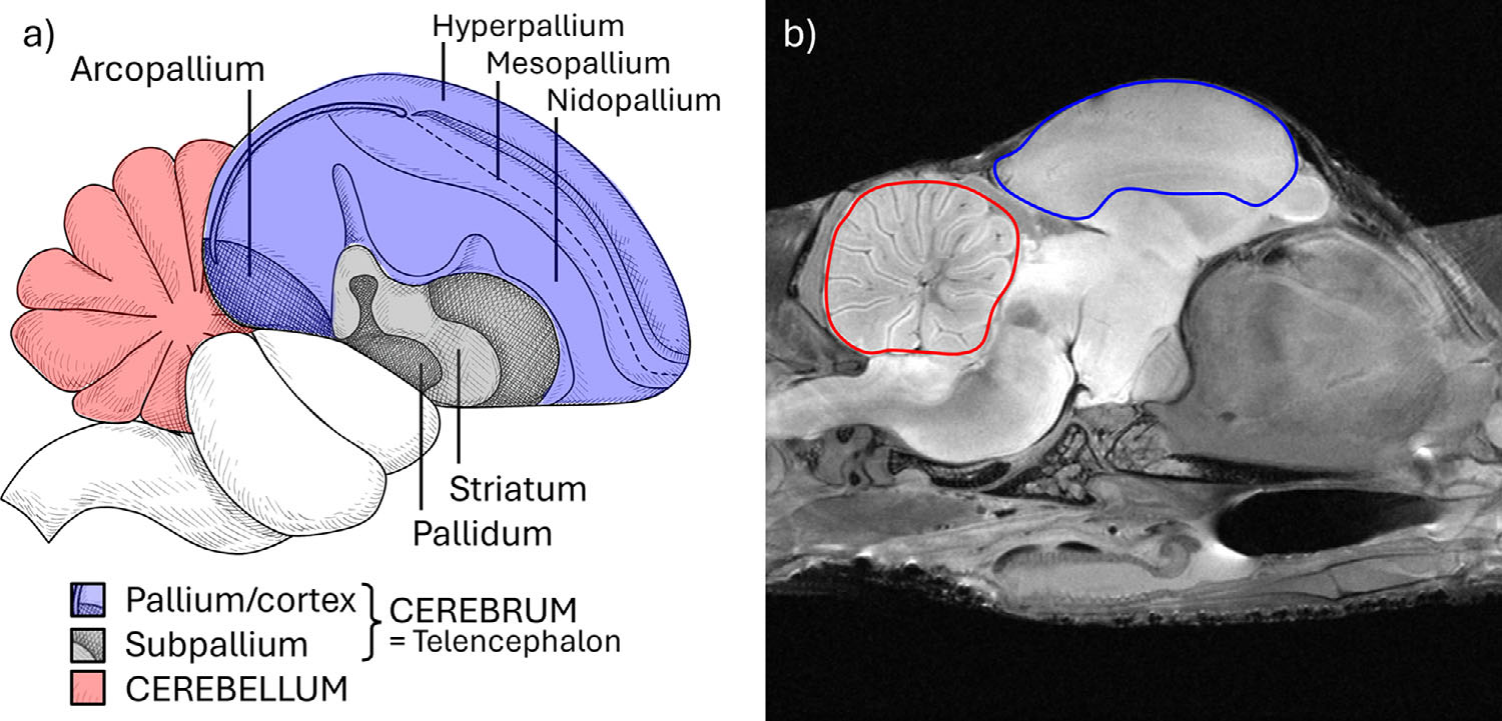
(a) Schematic representation of the chick embryo brain highlighting the two main regions analyzed in this study, including the pallial subdivisions and subpallial structures. (b) Representative MRI image showing the ROIs used for image analysis: the cerebellum (red) and the pallium (blue). These ROIs were consistently applied for both T1 quantification and CEST spectral acquisition.

Following Mn^2^^+^ administration, a substantial reduction in *T*_1_ was observed in both cerebellum and pallium, decreasing from 1.67 ± 0.05 to 1.20 ± 0.05 s in the cerebellum and from 1.78 ± 0.08 to 1.29 ± 0.06 s in the pallium, corresponding to an approximate 28% reduction.

As apparent from the *T*_1_ values reported in Figure 2 and from the corresponding *T*_1_‐weighted images, Mn^2^^+^ is initially largely confined to the vascular compartment, with minimal penetration into the brain parenchyma shortly after injection. At these early time points, the observed signal changes are dominated by the intravascular pool, with no appreciable tissue‐associated *T*_1_ shortening. A clear transition occurs after approximately 3 h, when a progressive decrease in *T*_1_ becomes evident within both the cerebellum and pallium, indicating the onset of Mn^2^^+^ uptake into brain tissue. By 24 h post‐injection, *T*_1_ values stabilize, and the spatial contrast pattern in the images becomes homogeneous within the parenchyma, consistent with the establishment of a steady‐state distribution. This behavior suggests that vascular and extracellular Mn^2^^+^ has largely washed out, while intracellularly retained Mn^2^^+^ persists, reflecting intracellular accumulation. On this basis, all CEST measurements were performed 24 h after Mn^2^^+^ administration to ensure that the detected contrast predominantly arises from intracellular Mn^2^^+^, minimizing contributions from vascular and extracellular compartments [15, 23].

**FIGURE 2:**
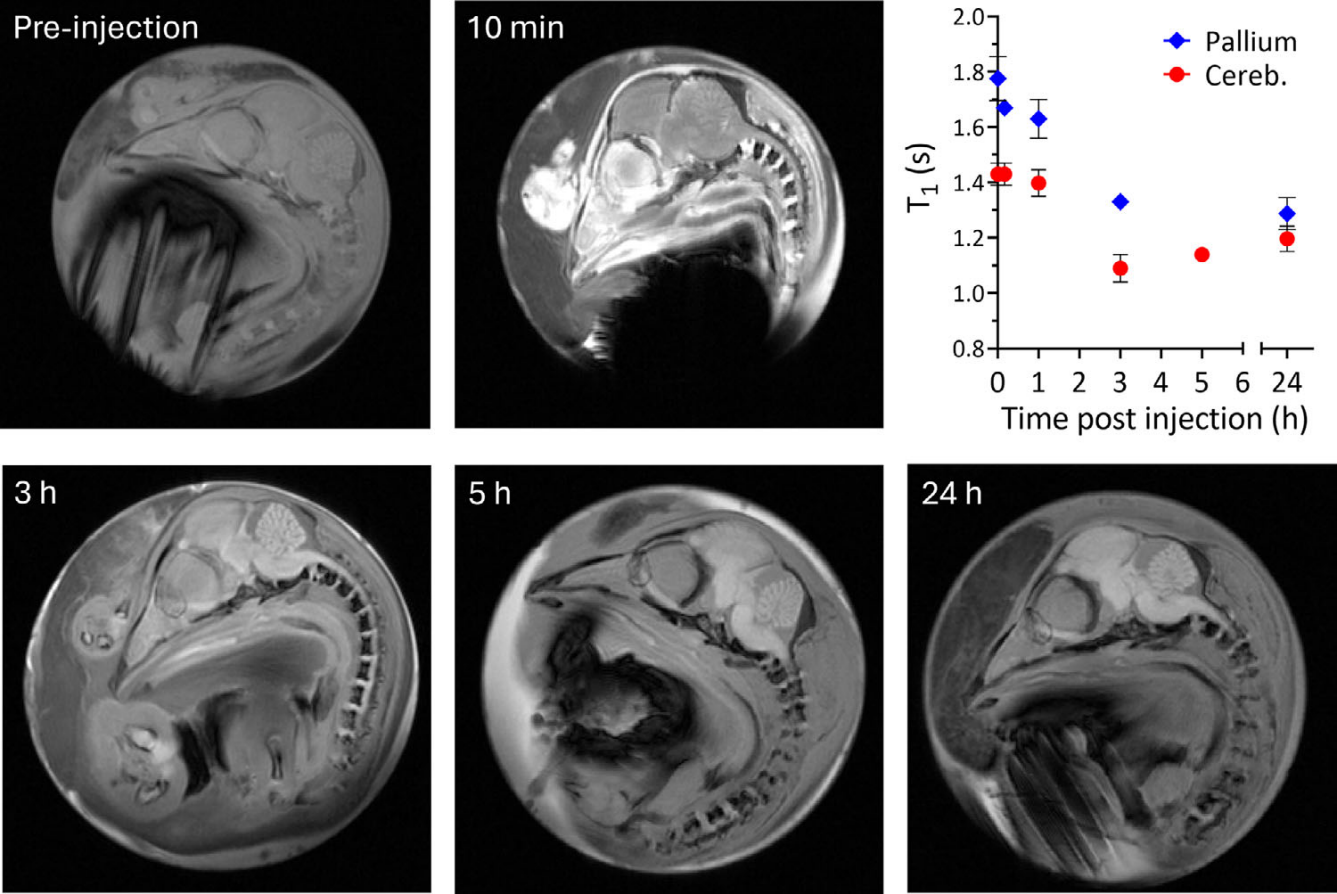
*T*₁‐weighted MRI of chick embryos in ovo acquired before and at different time points following Mn^2^^+^ administration (pre‐injection, 10 min, 3, 5, and 24 h). The indicated time points refer to the duration of incubation after Mn injection; MRI data were acquired after the reported interval plus an additional 30 min cooling period at 4°C. T₁‐weighted images show the progressive redistribution of Mn^2^^+^, from an initial predominantly vascular signal enhancement at early time points to a more homogeneous parenchymal contrast at later stages. The upper‐right panel reports *T*₁ values measured in regions of interest in the pallium (blue diamonds) and cerebellum (red circles), corresponding to those used for Z‐spectra.

Although the embryos remained viable and showed no gross evidence of tissue damage, dedicated histological and molecular assessments of Mn‐induced toxicity were not performed. Potential subclinical effects of Mn^2^^+^ therefore cannot be excluded and should be investigated in future dose–response studies.

High‐resolution ex vivo MRI at 24 h post‐injection provides direct evidence of region‐ and cell‐specific Mn^2^^+^ accumulation within the chick brain (Figure 3). The images reveal a pronounced laminar organization in the cerebellum, with a well‐defined hyperintense band consistent with selective accumulation in Purkinje neurons, in agreement with previous reports assigning MEMRI contrast predominantly to this neuronal population [23]. At this spatial resolution, the cerebellar foliation and cortical layering are clearly resolved, highlighting the compartmentalized distribution of Mn^2^^+^ within the parenchyma. The sharp delineation of anatomical substructures and the persistence of contrast after extracellular washout further support that Mn^2^^+^ is predominantly retained intracellularly within neurons. In addition to the cerebellum, detectable signal enhancement is observed in the telencephalon and brainstem, indicating broader cellular uptake.

**FIGURE 3:**
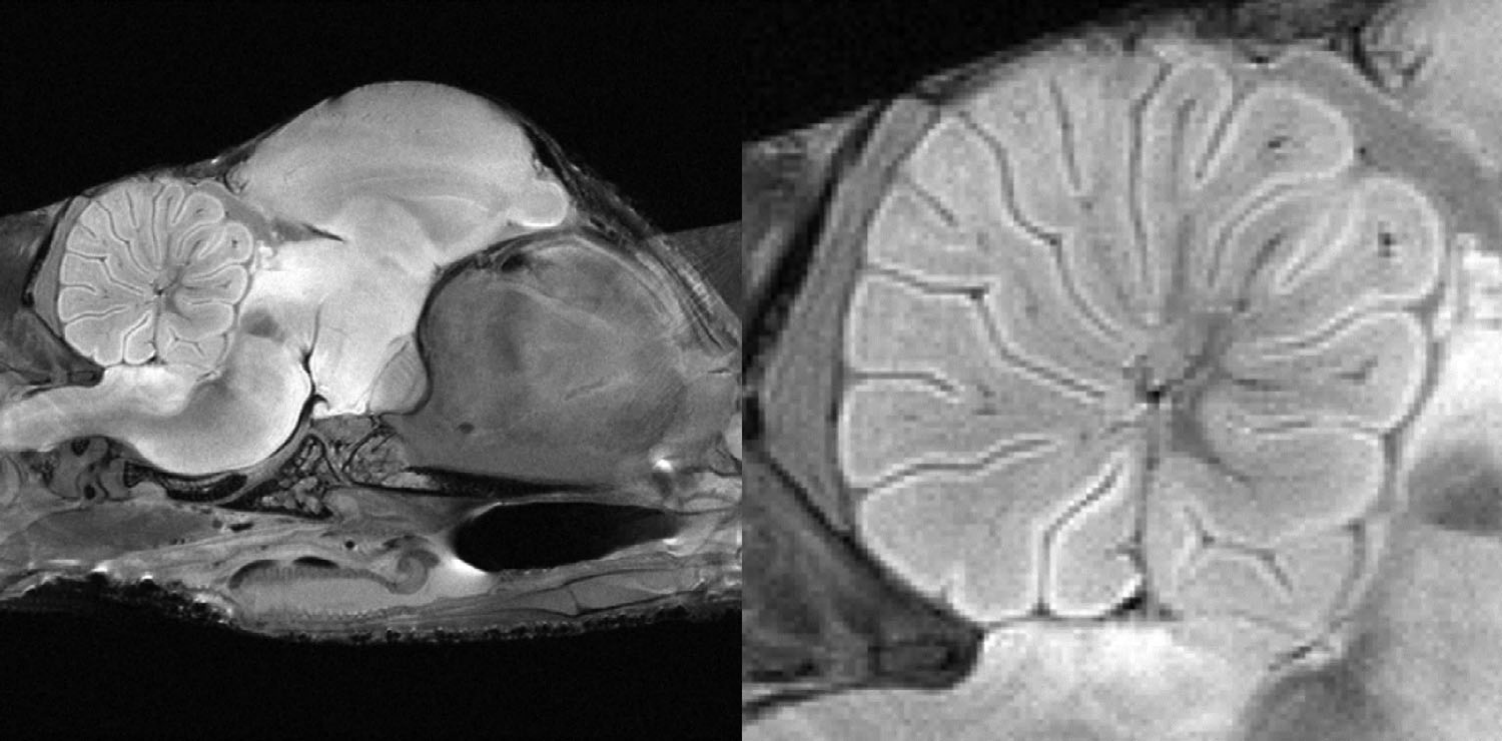
High‐resolution *T*₁‐weighted MRI of the chick brain acquired ex vivo 24 h after Mn^2^^+^ administration. FLASH images were obtained at 60 µm isotropic resolution using a quadrature volume coil (23 mm inner diameter; FOV 22 mm). The excised head was sealed in a vacuum plastic enclosure for imaging. The images reveal pronounced laminar contrast in the cerebellum and region‐specific Mn^2^^+^ accumulation across brain structures, consistent with predominant neuronal uptake.

CEST Z‐spectra acquired from the cerebellum exhibited the expected composite profile arising from multiple exchanging proton pools (Figure 4a), including amine and hydroxyl groups, predominantly arising from myo‐inositol and choline (0.8–1 ppm). Additional contributions were observed from amine protons associated with creatine and phosphocreatine (∼2 ppm), GABA (∼2.75 ppm), glutamate and glutamine (∼3 ppm), and amide proton transfer originating from proteins and peptides (∼3.5 ppm), a peak attributable to N‐acetyl aspartate was detected at ∼3.7 ppm, along with contributions from serotonergic system protons (∼5.4 ppm) and NOE effects at negative frequency offsets. Figure SI.2 presents an idealized and enhanced CEST spectrum highlighting the various exchanging CEST pools and the two NOE contributions.

**FIGURE 4:**
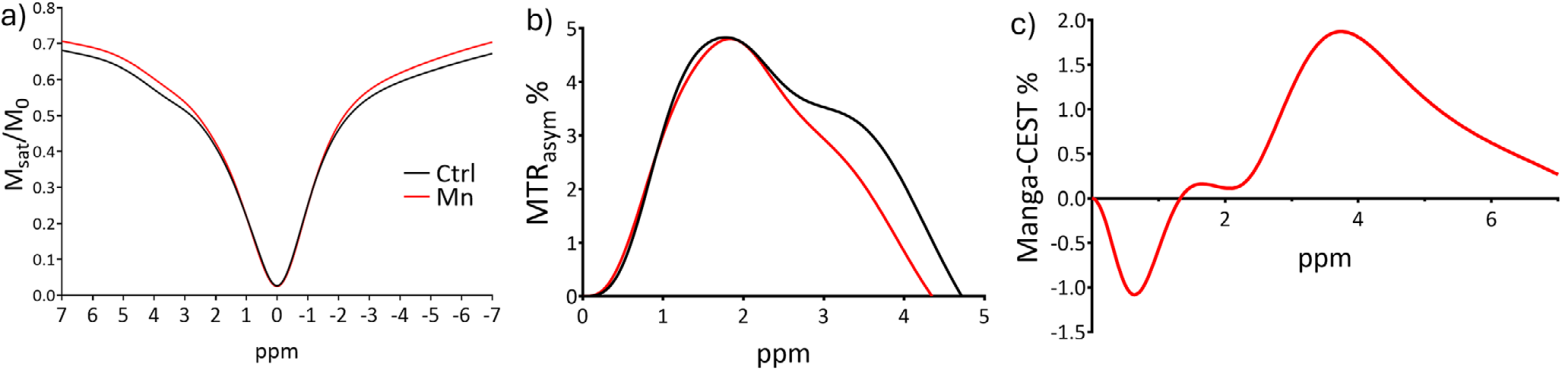
(a) Z‐spectra acquired from chick embryos in control conditions (Ctrl) (*n* = 5) and following manganese administration (Mn^2^^+^) (*n* = 5) for the cerebellum ROI. (b) MTR_asym_ % spectrum in the frequency range of interest. (c) MangaCEST % spectrum obtained by subtracting the control Z‐spectrum from those acquired after Mn administration, highlighting Mn‐induced contrast differences.

The spectra shown in Figure 4 represent the average of acquisitions obtained from five chicks per group (*n* = 5). Following Mn^2^^+^ administration, a clear and reproducible modification of the Z‐spectrum was observed, characterized by a frequency‐dependent attenuation of specific spectral features (Figure 4a). A reduction in signal intensity was observed in the frequency range between approximately 3 and 4 ppm, a region commonly associated with contributions from metabolites enriched in neuronal compartments, including N‐acetyl aspartate and glutamate‐weighted signals. Importantly, these changes were not uniform across the spectrum, indicating that the effect cannot be attributed solely to a global scaling of signal intensity.

This attenuation is more clearly visualized in the asymmetry spectrum derived from the two MTRasym spectra, with or without Mn (Figure 4b), where a reduction in the component centered in the 3–4 ppm range is evident. This feature is consistent with contributions from intracellular exchangeable proton pools that include N‐acetyl aspartate, a well‐established spectroscopic marker of neuronal integrity. While the asymmetry analysis enhances the visibility of this effect, it should be noted that this spectral region may include overlapping contributions from multiple exchangeable pools.

To further characterize this effect, differential spectra were computed by subtracting, at each positive frequency offset, the control Z‐spectrum from that acquired following Mn^2^^+^ administration, the MangaCEST‐spectrum reported in Figure 4c. This pseudo‐CEST spectrum highlights the Mn‐sensitive component of the CEST signal. Notably, the prominence of the differential signal in the 3–4 ppm range is consistent with the expected distribution of neuronal metabolites, which are present at high intracellular concentrations and are therefore more sensitive to perturbations affecting the intraneuronal water pool. Given the strong evidence for neuronal enrichment of Mn^2^^+^ in the cerebellum, it is reasonable to interpret these Mn‐sensitive components as being enriched for neuronal‐associated contributions, although not exclusively specific to them.

The broad 3–4 ppm difference cannot be assigned uniquely to NAA and likely contains overlapping contributions from amide, amine, and metabolite pools. In addition, Mn‐induced changes in MT and NOE‐related cross‐relaxation may contribute to the observed lineshape. Because the present model did not include an explicit NOE pool, such contributions cannot be excluded.

The Mn‐induced attenuation of the CEST signal is expected to arise not only from changes in water relaxation but also from altered relaxation properties of intracellular exchanging proton pools. In particular, shortening of the *T*_2_ of the exchanging pools is expected to reduce saturation transfer efficiency and thereby contribute to the observed MangaCEST contrast.

For comparison, Figure 5 reports the Z‐spectra and corresponding differential spectra obtained from the pallium. Differences between the MangaCEST spectra of the pallium and cerebellum may reflect differences in structural organization and underlying neurochemical composition. In the pallium, in addition to attenuation of features in the 3–4 ppm range, a reduction in signal is also observed around ∼3 ppm. This spectral region is commonly associated with glutamate‐weighted contributions [26, 27], which are expected to be more prominent in pallial tissue [28, 29].

**FIGURE 5:**
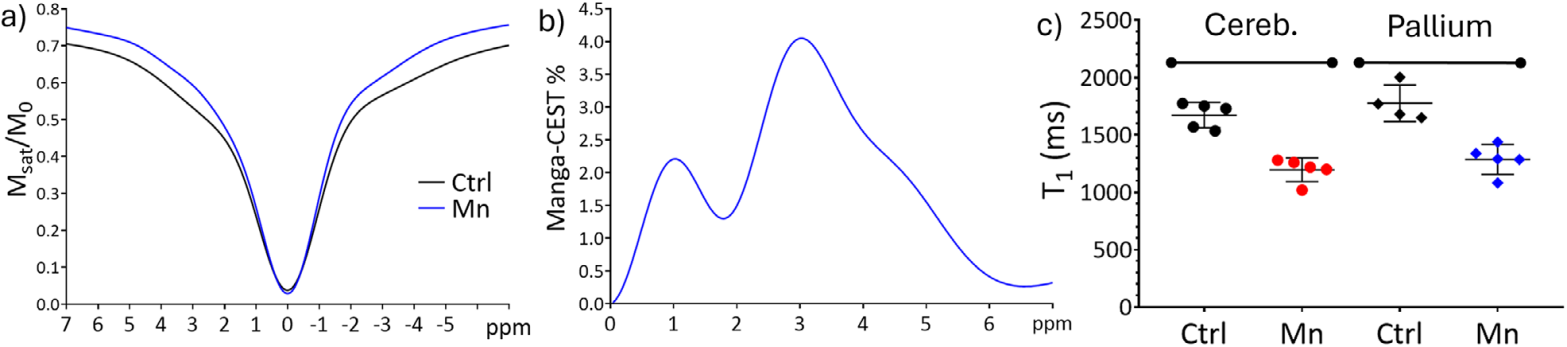
(a) Z‐spectra acquired from chick embryos in control conditions (Ctrl) (*n* = 5) and following manganese administration (Mn^2^^+^) (*n* = 5) for the pallium. (b) MangaCEST % spectrum calculated as the difference between the Z‐spectra acquired before and after Mn^2^^+^ administration. (c) Quantification of relaxation times measured within the corresponding ROIs (cerebellum and pallium), reported for both experimental conditions.

To assess whether the observed alterations in the CEST spectra could be explained solely by nonspecific Mn‐induced T_1_ shortening, numerical simulations of the Z‐spectrum were performed [30] (see Supporting Information). The results indicate that changes in *T*_1_ and *T*_2_, together with associated direct water saturation effects, can significantly influence the low‐offset region of the spectrum (within ∼2 ppm), primarily through small variations in the effective linewidth of the water resonance.

Specifically, water linewidth changes in the range of 2%–3% resulted in ±2% changes in the MangaCEST signal in this region, consistent with the trends observed experimentally in both cerebellum and pallium (particularly apparent in the negative dip in the low‐frequency end of the pseudo‐CEST spectrum of the cerebellum in Figure 4c). However, modulation of relaxation parameters alone was insufficient to reproduce the full pattern of spectral changes, particularly in the high‐frequency range. This finding supports the interpretation that compartment‐specific attenuation of metabolite‐associated contributions is required to account for the observed MangaCEST contrast.

Rallapalli et al. [23] support a strongly neuron‐dominant Mn^2^^+^ uptake in the cerebellar Purkinje cell layer under their experimental conditions, consistent with our own findings (Figure 3). Given that Mn^2^^+^ entry is closely coupled to calcium influx through voltage‐gated pathways—a mechanism broadly conserved across excitable neuronal populations—it is plausible that a similar neuron‐weighted uptake may also occur in other brain regions. Voltage‐gated calcium channels are the primary mediators of depolarization‐induced calcium entry in neurons [31], whereas glial calcium signaling relies predominantly on receptor‐operated and intracellular pathways [32].

However, in contrast to the cerebellum, the nonlaminar cytoarchitecture of the pallium does not allow clear spatial segregation of manganese accumulation between neuronal and glial components in the MR images, and the effects of manganese cannot be unambiguously attributed to neuronal versus glial compartments. Consequently, a definitive attribution of the CEST features observed in the pallium to a dominant neuronal compartment remains speculative, and further validation is required.

Beyond its potential to probe compartmental contributions, MangaCEST may offer additional avenues for investigating brain function. Because Mn^2^^+^ accumulation is coupled to calcium influx during cellular activation, the perturbation underlying MangaCEST may reflect aspects of neuronal and glial activity integrated over time. This raises the possibility that MangaCEST could be extended toward an activity‐weighted form of molecular spectroscopy, in which CEST contrast is influenced not only by cellular composition but also by the functional engagement of different cell populations. In principle, MangaCEST could be combined with localized manganese administration in tract‐tracing paradigms [33, 34] to enhance the contribution of cells engaged in specific functional pathways. Realizing this potential will require controlled studies designed to disentangle activity‐dependent effects from baseline differences in tissue composition and relaxation properties.

The principles underlying MangaCEST may also be relevant to organs other than the brain. Mn‐enhanced MRI has been extensively applied in cardiac imaging [35, 36], where Mn^2^^+^ uptake occurs via voltage‐gated calcium channels and provides a surrogate measure of myocardial calcium handling and viability. In this context, Mn‐induced modulation of CEST contrast could, in principle, enable activity‐ and viability‐weighted molecular interrogation of the myocardium, although this possibility remains to be explored.

In conclusion, we introduce MangaCEST as a strategy to modulate the contribution of intracellular compartments to the CEST MRI signal using a paramagnetic perturbation. By exploiting the preferential accumulation of manganese in excitable cells and its effect on water relaxation, this approach enables differential interrogation of CEST contrast in vivo.

Using the cerebellum as a model system, in which Mn^2^^+^ is known to accumulate preferentially in neurons, we show that Mn^2^^+^ administration induces reproducible, frequency‐dependent changes in the CEST Z‐spectrum. The resulting differential spectra reveal Mn‐sensitive components consistent with contributions from intracellular metabolite pools associated with neuronal compartments.

These findings support MangaCEST as a promising approach for probing compartment‐weighted molecular information in vivo. Future work will be required to extend this framework beyond the favorable model systems used in this proof‐of‐concept study, to quantify and correct for relaxation‐driven confounds, and to validate the cellular origin of the observed contrast using complementary approaches. Nevertheless, MangaCEST provides a novel strategy to improve the biological interpretability of CEST MRI and represents a significant step toward improved cellular specificity in molecular imaging of the brain.

## Author Contributions

**Eleonora Cavallari**: conceptualization, writing ‐ original draft, methodology, validation, software, formal analysis, data curation, investigation, project administration. **Valeria Bitonto**: methodology, writing ‐ review and editing, investigation. **Morgana Giachello**: writing ‐ review and editing, methodology, investigation. **Angelo Bifone**: conceptualization, funding acquisition, supervision, resources, methodology, validation, writing ‐ original draft, project administration, investigation.

## Conflicts of Interest

The authors declare no conflicts of interest.

## Supporting information



Additional references are provided in the Supporting Information [37, 38, 39, 40, 41, 42, 43, 44, 45]. **Supporting File**: anie74607‐sup‐0001‐SuppMat.docx.

## Data Availability

The data that support the findings of this study are available from the corresponding author upon reasonable request.
