# Supplementary material for: Toward Cell‐Specific Molecular MRI: Modulating Chemical Exchange Saturation Transfer Contrast With Manganese
Source: Angew Chem Int Ed Engl. 2026 Sep 9;65(43):e1291856. doi: 10.1002/anie.1291856 (PMC13676406; doi:10.1002/anie.1291856)
Supplement: Supplementary file 1 [file ANIE-65-e1291856-s001.docx]

Toward Cell-Specific Molecular MRI: Modulating Chemical Exchange Saturation Transfer Contrast with Manganese

Eleonora Cavallari*^[a]^, Valeria Bitonto^[a]^, Morgana Giachello^[a]^, Angelo Bifone^[a]^

[a]	Department of Molecular Biotechnology and Health Sciences
University of Torino
Piazza Nizza 44 bis, 10126, Torino, Italy

Supporting Information

*Adjustment of Developmental Observation Timing According to Chicken Breed*

Fresh fertilized eggs of Antwerpse Baardkriel (Gallus gallus domesticus) were incubated at 37.7 °C and 60% humidity (incubator: FIEM CIP CIP 28 MINILED). This study used pre-hatch avian embryos (chicken embryos), which do not require specific authorization for animal experimentation under Italian regulations.

The Antwerpse Baardkriel hens used in this study lay relatively small eggs (mean diameter 3.5–3.7 cm), compared to those of standard commercial breeds (typically 5–6 cm). Based on our observations, embryos from this breed tend to hatch slightly earlier (around embryonic day 20), with developmental progression appearing modestly accelerated relative to larger-egg breeds. This phenomenon is consistent with previous reports indicating that egg size can influence the rate of embryonic development and the timing of hatching, with smaller eggs generally associated with a slight advancement in developmental milestones. Accordingly, embryonic day 17 (E17) in this model is developmentally comparable to embryonic day 18 (E18) reported in studies using larger eggs.

*Manganese administration and embryos preparation for MRI*

The Mn-treated chick group (n = 5 embryos) received a dose of Mn²⁺ (28.5 mg/kg) via injection of a 100 mM Mn²⁺ solution in bicine buffer into the chorioallantoic vein. The chorioallantoic membrane, which becomes highly vascularized between embryonic day 7 and 12, was used for systemic delivery of manganese via injection into chorioallantoic veins.

Mn²⁺ dissolved in PBS is highly pH-sensitive, leading to the formation of amorphous precipitated crystals at physiological pH; therefore, it was dissolved in bicine buffer, which provides greater stability from both chemical and relaxometric perspectives^[37]^. The 28.5 mg/kg dose was adjusted to the chick’s body weight at embryonic day 17 (E17), considering an embryonated egg weight of approximately 27 ± 2 g and an embryo weight of 13–15 g. Prior to imaging, eggs were cooled at 4 °C for 30 minutes to induce mild hypothermia and reduce motion artifacts^[38,39]^.

*Image analysis*

The region of interest (ROI) was manually segmented to include the chick pallium and cerebellum on the anatomical reference image of each animal (Figure 1b) using the Fiji–ImageJ software (https://imagej.nih.gov/ij/download.html and https://imagej.net/Fiji/Downloads). Signal intensity and standard deviation were then extracted and imported into OriginPro 2016. Individual Z-spectra were normalized to the signal acquired at ±100 ppm and corrected for B₀ inhomogeneities. Five Z-spectra for each experimental group (Ctrl and Mn²⁺) were averaged to obtain the final Z-spectrum (Figure 4a for the cerebellum and Figure 5a for the pallium).

Segmentation for Z-spectra extraction and T₁ quantification was based on high-resolution T₂-weighted anatomical and T₁-weighted FLASH images (0.18 × 0.18 mm), and subsequently transferred to the lower-resolution datasets (T₁-VTR: 0.48 × 0.48 mm; CEST: 0.36 × 0.36 mm).

Quantitative T₁ maps were acquired using a saturation recovery RARE sequence with multiple repetition times (VTR-RARE). Representative examples of T₁ maps for a control chick embryo and an Mn-injected embryo are shown in Figure S.I.1.

T₁-weighted images were acquired with an in-plane resolution of 0.48 × 0.48 mm, using seven TR values on a single sagittal slice covering the regions of interest (cerebellum and telencephalon), with a total acquisition time of approximately 7 minutes. For each embryo, T₁ mapping was performed prior to CEST acquisition to verify Mn²⁺ accumulation and ensure consistency with the expected contrast enhancement before proceeding with further acquisitions and analysis. The T₁ values obtained from these measurements are reported in Figure 4c in the main text.

**Figure S.I.1** Representative T₁ of chick embryos: a) control embryo (Ctrl) and b) embryo imaged 24 h after intravenous injection of Mn²⁺ (28.5 mg kg⁻¹). The post-injection image shows a marked decrease of T₁ values in cerebellum and telencephalon, consistent with Mn²⁺ accumulation these specific anatomical regions.

*Interpretation of the in vivo CEST Z-Spectrum*

CEST spectra (also referred to as Z-spectra) reflect the combined effects of multiple physical and physiological processes, and their interpretation requires both an understanding of the underlying exchange mechanisms and careful control of acquisition parameters. Several experimental and biological factors influence the shape and amplitude of the Z-spectrum, including magnetic field strength, B₁ inhomogeneity, radiofrequency (RF) saturation power and duration, pH and temperature, as well as longitudinal (T₁) and transverse (T₂) relaxation times. In addition, non-specific contributions such as direct water saturation and magnetization transfer (MT) from semi-solid macromolecules can significantly affect the observed signal.

In a typical Z-spectrum, the signal is plotted as a function of the frequency offset of the saturation pulse relative to the water resonance (defined as 0 ppm). Unlike conventional magnetic resonance spectroscopy (MRS), where 0 ppm represents a spectral peak, in CEST this frequency corresponds to the point of maximum direct saturation of bulk water, resulting in the lowest signal intensity.

Exchangeable protons in endogenous metabolites give rise to CEST effects at positive frequency offsets (conventionally displayed on the left-hand side of the spectrum), including amide (~3.5 ppm), amine (~2–3 ppm), and hydroxyl (~0.5–1 ppm) groups. These contributions are often collectively referred to as the “CEST effect” and are sensitive to molecular environment and exchange rates. At negative frequency offsets (right-hand side), the spectrum is dominated by nuclear Overhauser enhancement (NOE) effects, which arise primarily from aliphatic protons in mobile macromolecules and lipids through cross-relaxation mechanisms.

**Figure S.I.2** Representative CEST Z-spectrum highlighting the main exchanging proton pools contributing to the brain CEST signal; negative frequency offsets highlight the NOE effects, and the dashed line indicates the magnetization transfer (MT) background.

The resulting Z-spectrum therefore represents a superposition of multiple components: specific chemical exchange effects from metabolites, NOE contributions, direct water saturation, and a broad MT background. As a consequence, individual spectral features cannot always be uniquely assigned to a single molecular species, and their interpretation must be considered in the context of these overlapping contributions.

Figure S.I.2 illustrates a representative Z-spectrum highlighting the principal exchanging proton pools contributing to brain CEST contrast. The relative positions of these features reflect their chemical shift offsets from water, while their amplitudes depend on both exchange dynamics and experimental conditions.

*MangaCEST simulation*

To further investigate the influence of relaxation times on the MangaCEST contrast (defined as the difference between Z-spectra), the simulated MangaCEST spectrum was calculated as the point-by-point difference between the simulated Z-spectrum obtained in the presence of Mn²⁺ and the corresponding control Z-spectrum using a MATLAB-based CEST simulation framework (Zaiss et al., available at http://www.cest-sources.org).

The model included three exchanging proton pools: bulk water, a semi-solid macromolecular pool representing white matter, and a solute pool responsible for the CEST effect. Relaxation times, exchange rates, and pool fractions were selected based on values measured in this study or reported in the literature (Table 1). In particular, the longitudinal relaxation time of water (T₁) was set to the *in vivo* values measured in chick embryos, while transverse relaxation times (T₂), magnetization transfer (MT) parameters and data for a representative CEST pool were derived from literature.

Mn²⁺ exhibits high transverse relaxivity, which is strongly modulated by the local physicochemical environment, including compartmentalization, microstructural heterogeneity, and water mobility. To account for these effects, a 15% reduction in T₂ was assumed for the cerebellum, consistent with increased intracellular Mn²⁺ accumulation and restricted water diffusion. A smaller reduction (5%) was assumed for the pallium, reflecting its lower cellular density and higher structural heterogeneity.

***Figure S.I.3*** *Simulated Z-spectra and corresponding MangaCEST pseudo-spectra illustrating the effect of Mn-induced relaxation changes. Top panels: Z-spectra simulated in the absence and presence of Mn²⁺ for representative relaxation conditions, showing the influence of T₁ and T₂ modulation on direct water saturation and spectral lineshape. Bottom panels: corresponding MangaCEST spectra, calculated as the point-by-point subtraction of the control Z-spectrum from the Mn-treated Z-spectrum. The simulations highlight a pronounced sensitivity of the low-frequency region (≤ ~2 ppm) to relaxation-induced linewidth variations, while failing to reproduce the full spectral changes observed experimentally, particularly in the 3.5–4 ppm range associated with metabolite contributions. a) and c) simulation using the parameter set defined for the cerebellum; b) and d) simulation using the parameter set defined for the pallium; see Table 1.*

Simulated Z-spectra and corresponding MangaCEST difference spectra are shown in Figure S.I.3. The simulations indicate that Mn-induced changes in relaxation parameters significantly affect direct water saturation and spectral line shape. In particular, subtraction of control and Mn-affected spectra result in pronounced differences in the low-frequency region (≤ 2 ppm), where the signal is highly sensitive to relaxation-induced linewidth variations.

Quantitatively, water linewidth changes of approximately 2–3% produced variations of about ±2% in the MangaCEST signal in this region, in agreement with experimental observations in both cerebellum and pallium. These results indicate that the low-frequency portion of the MangaCEST spectrum is largely dominated by relaxation effects.

Simulations incorporating the experimentally measured water T₁ shortening and estimated changes in water T₂ reproduced these relaxation-driven effects close to the water resonance. **However, modulation of relaxation parameters alone was insufficient to reproduce the full experimental difference spectrum, particularly in the 3–4 ppm region, which is likely to reflect the combined effects of water relaxation, spillover, magnetization transfer (MT), and modulation of intracellular exchanging proton pools.** These findings support the interpretation that compartment-specific attenuation of metabolite-associated CEST signals contributes to the observed MangaCEST contrast.

To better understand the underlying relaxation mechanisms, we performed additional simulations including Mn-induced changes in both T₁ and T₂ of the exchanging solute pools. While these simulations are not reported here, they confirmed that shortening of the T₂ of the exchanging protons has a substantially larger effect on the CEST spectrum than the corresponding changes in T₁. This supports the interpretation that the Mn-induced attenuation of the CEST signal arises not only from changes in water relaxation but also from altered relaxation properties of intracellular exchanging proton pools. In particular, shortening of the T₂ of the exchanging pools is expected to reduce saturation transfer efficiency and to contribute substantially to the observed MangaCEST contrast.

**Table 1.** Summary of parameters used for the CEST simulations. Reported values include relaxation times, exchange rates, and pool fractions, obtained either from measurements in this work or from literature sources, as specified. The Mn-associated T₂ values used in the simulations should therefore be regarded as estimates introduced to explore the sensitivity of the Z-spectrum to plausible transverse relaxation changes, rather than as experimentally determined values.

| Parameter | Assigned value | Source |
| --- | --- | --- |
| T_1,H2O,Ctrl_  cerebellum/pallium | 1672 ms/1776 ms | Measured in the cerebellum and pallium of control embryos in this study |
| T_2,H2O,Ctrl_  cerebellum/pallium | 40 ms/60 ms | ^[9,40,41]^ |
| T_1,H2O,Mn_  cerebellum/pallium | 1196 ms/1287 ms | Measured in the cerebellum and pallium of control embryos in this study |
| T_2,H2O,Mn_  cerebellum/pallium | 38 ms/51 ms | The reduction in T₂ was estimated based on the relaxivity effect (r₂) reported in the literature ^[9]^ |
| f_APT_ | 0.000655 | ^[42]^ |
| k_APT_ | 22 Hz | ^[42]^ |
| T_1,APT_ | 1 s | ^[7,43]^ |
| T_2,APT_ | 10 ms | ^[7,43]^ |
| f_MT_ | 5 % | ^[44]^ |
| k_MT_ | 24 Hz | ^[44]^ |
| T_1,MT_ | 1 s | ^[7,45]^ |
| T_2,MT_ | 10 us | ^[45]^ |

[1]	S. D. Wolff, R. S. Balaban, “Magnetization transfer contrast (MTC) and tissue water proton relaxation *in vivo*” *Magn. Reson. Med.* **1989**, *10*, 135–144.

[2]	V. Guivel-Scharen, T. Sinnwell, S. D. Wolff, R. S. Balaban, “Detection of Proton Chemical Exchange between Metabolites and Water in Biological Tissues” *Journal of Magnetic Resonance* **1998**, *133*, 36–45.

[3]	K. M. Ward, A. H. Aletras, R. S. Balaban, “A New Class of Contrast Agents for MRI Based on Proton Chemical Exchange Dependent Saturation Transfer (CEST)” *Journal of Magnetic Resonance* **2000**, *143*, 79–87.

[4]	P. C. M. Van Zijl, N. N. Yadav, “Chemical exchange saturation transfer (CEST): What is in a name and what isn’t?” *Magn. Reson. Med.* **2011**, *65*, DOI 10.1002/mrm.22761.

[5]	K. M. Jones, A. C. Pollard, M. D. Pagel, “Clinical applications of chemical exchange saturation transfer (CEST) MRI” *Journal of Magnetic Resonance Imaging* **2018**, *47*, DOI 10.1002/jmri.25838.

[6]	A. N. Dula, E. M. Asche, B. A. Landman, E. B. Welch, S. Pawate, S. Sriram, J. C. Gore, S. A. Smith, “Development of chemical exchange saturation transfer at 7T” *Magn. Reson. Med.* **2011**, *66*, 831–838.

[7]	M. Zaiss, P. Bachert, “Exchange‐dependent relaxation in the rotating frame for slow and intermediate exchange – modeling off‐resonant spin‐lock and chemical exchange saturation transfer” *NMR Biomed.* **2013**, *26*, 507–518.

[8]	R. A. Cloyd, S. A. Koren, J. F. Abisambra, “Manganese-Enhanced Magnetic Resonance Imaging: Overview and Central Nervous System Applications With a Focus on Neurodegeneration” *Front. Aging Neurosci.* **2018**, *10*, DOI 10.3389/fnagi.2018.00403.

[9]	A. C. Silva, J. H. Lee, I. Aoki, A. P. Koretsky, “Manganese‐enhanced magnetic resonance imaging (MEMRI): methodological and practical considerations” *NMR Biomed.* **2004**, *17*, 532–543.

[10]	R. E. London, G. Toney, S. A. Gabel, A. Funk, “Magnetic resonance imaging studies of the brains of anesthetized rats treated with manganese chloride” *Brain Res. Bull.* **1989**, *23*, 229–235.

[11]	E. Cavallari, E. Lorenzi, E. Di Gregorio, G. Ferrauto, S. Aime, G. Vallortigara, A. Bifone, “In vivo assessment of the influence of general anesthetics on transmembrane water cycling in the brain” *Journal of Cerebral Blood Flow and Metabolism* **2025**, *45*, 977–988.

[12]	E. Di Gregorio, C. Papi, L. Conti, A. Di Lorenzo, E. Cavallari, M. Salvatore, C. Cavaliere, G. Ferrauto, S. Aime, “A Magnetic Resonance Imaging-Chemical Exchange Saturation Transfer (MRI-CEST) Method for the Detection of Water Cycling across Cellular Membranes” *Angew. Chem. Int. Ed.* **2024**, *63*, DOI 10.1002/anie.202313485.

[13]	O. Natt, T. Watanabe, S. Boretius, J. Radulovic, J. Frahm, T. Michaelis, “High-resolution 3D MRI of mouse brain reveals small cerebral structures in vivo” *J. Neurosci. Methods* **2002**, *120*, 203–209.

[14]	A. C. Silva, J. H. Lee, C. W.-H. Wu, J. Tucciarone, G. Pelled, I. Aoki, A. P. Koretsky, “Detection of cortical laminar architecture using manganese-enhanced MRI” *J. Neurosci. Methods* **2008**, *167*, 246–257.

[15]	J. H. Lee, A. C. Silva, H. Merkle, A. P. Koretsky, “Manganese‐enhanced magnetic resonance imaging of mouse brain after systemic administration of MnCl _2_ : Dose‐dependent and temporal evolution of *T* _1_ contrast” *Magn. Reson. Med.* **2005**, *53*, 640–648.

[16]	R. Zhang, Z. Fa, Y. Liu, H. Sun, P. Li, S. Li, X. Wang, H. Lei, X. Jiang, “Dynamic MRI of rat brain following manganese administration through the internal carotid artery” *Neurol. Res.* **2014**, *36*, 679–686.

[17]	S. Tambalo, A. Daducci, S. Fiorini, F. Boschi, M. Mariani, M. Marinone, A. Sbarbati, P. Marzola, “Experimental protocol for activation‐induced manganese‐enhanced MRI (AIM‐MRI) based on quantitative determination of Mn content in rat brain by fast *T* _1_ mapping” *Magn. Reson. Med.* **2009**, *62*, 1080–1084.

[18]	T. L. Roth, D. Nayak, T. Atanasijevic, A. P. Koretsky, L. L. Latour, D. B. McGavern, “Transcranial amelioration of inflammation and cell death after brain injury” *Nature* **2014**, *505*, 223–228.

[19]	C. H. Liu, H. E. D’Arceuil, A. J. de Crespigny, “Direct CSF injection of MnCl _2_ for dynamic manganese‐enhanced MRI” *Magn. Reson. Med.* **2004**, *51*, 978–987.

[20]	E. Lorenzi, S. Tambalo, G. Vallortigara, A. Bifone, “Manganese-enhanced magnetic resonance imaging reveals light-induced brain asymmetry in embryo” *Elife* **2023**, *12*, DOI 10.7554/eLife.86116.

[21]	M. Aschner, M. Gannon, H. K. Kimelberg, “Manganese Uptake and Efflux in Cultured Rat Astrocytes” *J. Neurochem.* **1992**, *58*, 730–735.

[22]	K. V. R. Rao, M. D. Norenberg, “Manganese Induces the Mitochondrial Permeability Transition in Cultured Astrocytes” *Journal of Biological Chemistry* **2004**, *279*, 32333–32338.

[23]	H. Rallapalli, N. S. Bayin, H. Goldman, D. Maric, B. J. Nieman, A. P. Koretsky, A. L. Joyner, D. H. Turnbull, “Cell specificity of Manganese-enhanced MRI signal in the cerebellum” *Neuroimage* **2023**, *276*, DOI 10.1016/j.neuroimage.2023.120198.

[24]	E. D. Jarvis, J. Yu, M. V. Rivas, H. Horita, G. Feenders, O. Whitney, S. C. Jarvis, E. R. Jarvis, L. Kubikova, A. E. P. Puck, C. Siang‐Bakshi, S. Martin, M. McElroy, E. Hara, J. Howard, A. Pfenning, H. Mouritsen, C. Chen, K. Wada, “Global view of the functional molecular organization of the avian cerebrum: Mirror images and functional columns” *Journal of Comparative Neurology* **2013**, *521*, 3614–3665.

[25]	E. D. Jarvis, O. Güntürkün, L. Bruce, A. Csillag, H. Karten, W. Kuenzel, L. Medina, G. Paxinos, D. J. Perkel, T. Shimizu, G. Striedter, J. M. Wild, G. F. Ball, J. Dugas-Ford, S. E. Durand, G. E. Hough, S. Husband, L. Kubikova, D. W. Lee, C. V. Mello, A. Powers, C. Siang, T. V. Smulders, K. Wada, S. A. White, K. Yamamoto, J. Yu, A. Reiner, A. B. Butler, “Avian brains and a new understanding of vertebrate brain evolution” *Nat. Rev. Neurosci.* **2005**, *6*, 151–159.

[26]	B. Schmitz‐Abecassis, C. Najac, J. Plugge, M. J. P. van Osch, E. Ercan, “Investigation of metabolite correlates of CEST in the human brain at 7 T” *NMR Biomed.* **2024**, *37*, DOI 10.1002/nbm.5104.

[27]	F. Severo, N. Shemesh, “In-vivo Magnetic Resonance Imaging of GABA and Glutamate” **2020**.

[28]	Y. Xu, Z. Zhuang, H. Zheng, Z. Shen, Q. Gao, Q. Lin, R. Fan, L. Luo, W. Zheng, “Glutamate Chemical Exchange Saturation Transfer (GluCEST) Magnetic Resonance Imaging of Rat Brain With Acute Carbon Monoxide Poisoning” *Front. Neurol.* **2022**, *13*, DOI 10.3389/fneur.2022.865970.

[29]	A. T. J. Cember, R. P. R. Nanga, R. Reddy, “Glutamate‐weighted CEST (gluCEST) imaging for mapping neurometabolism: An update on the state of the art and emerging findings from *in vivo* applications” *NMR Biomed.* **2023**, *36*, DOI 10.1002/nbm.4780.

[30]	M. Zaiss, Z. Zu, J. Xu, P. Schuenke, D. F. Gochberg, J. C. Gore, M. E. Ladd, P. Bachert, “A combined analytical solution for chemical exchange saturation transfer and semi-solid magnetization transfer” *NMR Biomed.* **2015**, *28*, DOI 10.1002/nbm.3237.

[31]	B. A. Simms, G. W. Zamponi, “Neuronal Voltage-Gated Calcium Channels: Structure, Function, and Dysfunction” *Neuron* **2014**, *82*, 24–45.

[32]	A. Verkhratsky, M. Nedergaard, “Physiology of Astroglia” *Physiol. Rev.* **2018**, *98*, 239–389.

[33]	R. G. Pautler, A. C. Silva, A. P. Koretsky, “*In vivo* neuronal tract tracing using manganese‐enhanced magnetic resonance imaging” *Magn. Reson. Med.* **1998**, *40*, 740–748.

[34]	S.-W. Sun, B. Campbell, C. Lunderville, E. Won, H.-F. Liang, “Noninvasive Topical Loading for Manganese-Enhanced MRI of the Mouse Visual System” *Investigative Opthalmology & Visual Science* **2011**, *52*, 3914.

[35]	T. Singh, S. Joshi, L. E. Kershaw, M. R. Dweck, S. I. Semple, D. E. Newby, “Manganese‐Enhanced Magnetic Resonance Imaging of the Heart” *Journal of Magnetic Resonance Imaging* **2023**, *57*, 1011–1028.

[36]	N. B. Spath, G. Thompson, A. H. Baker, M. R. Dweck, D. E. Newby, S. I. K. Semple, “Manganese-enhanced MRI of the myocardium” *Heart* **2019**, *105*, 1695–1700.

[37]	C. A. Massaad, R. G. Pautler in *Methods in Molecular Biology*, Humana Press Inc., **2011**, pp. 145–174.

[38]	G. Winter, A. B. F. Koch, J. Löffler, F. Jelezko, M. Lindén, H. Li, A. Abaei, Z. Zuo, A. J. Beer, V. Rasche, “In vivo PET/MRI Imaging of the Chorioallantoic Membrane” *Front. Phys.* **2020**, *8*, DOI 10.3389/fphy.2020.00151.

[39]	Z. Zhou, Z. Chen, J. Shan, W. Ma, L. Li, J. Zu, J. Xu, “Monitoring brain development of chick embryos in vivo using 3.0 T MRI: subdivision volume change and preliminary structural quantification using DTI” *BMC Dev. Biol.* **2015**, *15*, 29.

[40]	R. A. de Graaf, P. B. Brown, S. McIntyre, T. W. Nixon, K. L. Behar, D. L. Rothman, “High magnetic field water and metabolite proton *T* _1_ and *T* _2_ relaxation in rat brain in vivo” *Magn. Reson. Med.* **2006**, *56*, 386–394.

[41]	Y. Boillat, P.-L. Bazin, K. O’Brien, M. J. Fartaria, G. Bonnier, G. Krueger, W. van der Zwaag, C. Granziera, “Surface-based characteristics of the cerebellar cortex visualized with ultra-high field MRI” *Neuroimage* **2018**, *172*, 1–8.

[42]	J. Zhou, J.-F. Payen, D. A. Wilson, R. J. Traystman, P. C. M. van Zijl, “Using the amide proton signals of intracellular proteins and peptides to detect pH effects in MRI” *Nat. Med.* **2003**, *9*, 1085–1090.

[43]	T. Jin, P. Wang, T. K. Hitchens, S.-G. Kim, “Enhancing sensitivity of pH-weighted MRI with combination of amide and guanidyl CEST” *Neuroimage* **2017**, *157*, 341–350.

[44]	G. J. Stanisz, E. E. Odrobina, J. Pun, M. Escaravage, S. J. Graham, M. J. Bronskill, R. M. Henkelman, “T _1_ , T _2_ relaxation and magnetization transfer in tissue at 3T” *Magn. Reson. Med.* **2005**, *54*, 507–512.

[45]	P. C. M. van Zijl, W. W. Lam, J. Xu, L. Knutsson, G. J. Stanisz, “Magnetization Transfer Contrast and Chemical Exchange Saturation Transfer MRI. Features and analysis of the field-dependent saturation spectrum” *Neuroimage* **2018**, *168*, 222–241.
